# Influence of Mechanical Properties on the Piezoelectric Response of UV-Cured Composite Films Containing Different ZnO Morphologies

**DOI:** 10.3390/polym15051159

**Published:** 2023-02-25

**Authors:** Donatella Duraccio, Pier Paolo Capra, Ambra Fioravanti, Giulio Malucelli

**Affiliations:** 1 Istituto di Scienze e Tecnologie per l’Energia e la Mobilità Sostenibili (STEMS), Consiglio Nazionale Delle Ricerche, Strada delle Cacce 73, 10135 Torino, Italy; 2 Istituto Nazionale di Ricerca Metrologica (INRIM), Strada delle Cacce 91, 10135 Torino, Italy; 3 Istituto di Scienze e Tecnologie per l’Energia e la Mobilità Sostenibili (STEMS), Consiglio Nazionale Delle Ricerche, Via Canal Bianco, 28, 44124 Ferrara, Italy; 4 Politecnico di Torino—Dipartimento di Scienza Applicata e Tecnologia, Viale Teresa Michel 5, 15121 Alessandria, Italy

**Keywords:** piezoelectric film, ZnO needles, ZnO flower-like structure, acrylic resin, mechanical properties

## Abstract

ZnO flower-like (ZFL) and needle (ZLN) structures were synthesized and embedded into UV-curable acrylic resin (EB), with the aim to study the effect of filler loading on the piezoelectric properties of the resulting composite films. The composites showed uniform dispersion of fillers within the polymer matrix. However, by increasing the filler amount, the number of aggregates increased, and ZnO fillers appeared not to be perfectly embedded in polymer film, indicating poor interaction with acrylic resin. The filler content increase caused an increase in glass transition temperature (T_g_) and a decrease in storage modulus in the glassy state. In particular, compared with pure UV-cured EB (T_g_ = 50 °C), 10 wt.% ZFL and ZLN presented T_g_ values of 68 and 77 °C, respectively. The piezoelectric response generated by the polymer composites was good when measured at 19 Hz as a function of the acceleration; the RMS output voltages achieved at 5 g were 4.94 and 1.85 mV for the composite films containing ZFL and ZLN, respectively, at their maximum loading levels (i.e., 20 wt.%). Further, the RMS output voltage increase was not proportional to the filler loading; this finding was attributable to the decrease in the storage modulus of the composites at high ZnO loading rather than the dispersion of filler or the number of particles on the surface.

## 1. Introduction

The process by which wind, thermal, solar, and mechanical sources are converted into electrical energy and stored for making self-powered systems is known as energy harvesting. Research and development on energy harvesting devices have rapidly grown in recent years, driven by quickly increasing energy consumption and further accelerated by concerns about carbon emissions from fossil fuel-based energy sources. Among the variety of renewable energy sources, mechanical energy (e.g., pressure, bending, stretching, and vibrational motions) is preferred over other types because of its ubiquity and accessibility from the surroundings [1,2]. The conversion of mechanical energy into electricity has been proposed by means of piezoelectric materials by several research groups [3,4].

A piezoelectric material is able to create a potential when subjected to mechanical stress due to the absence of a symmetry center in its crystals (dipole structure). More precisely, when vibration, tension, bending, or compression [5] results in a deformation of the dipole moment, a polarization phenomenon occurs, which is able to induce an external electric potential difference. There are two piezoelectric effects: the first is the so-called direct piezoelectric effect, which represents the material capability to convert mechanical strain into electricity. This effect can be exploited for designing sensors. The second one is the converse effect, which assesses that material capability to transform an applied electrical potential into mechanical energy (thus behaving as an actuator). Piezoelectric materials are ferroelectric systems in which the peculiar orientation of their molecular structure results in an electric dipole [6,7,8,9,10].

There already exist a number of excellent reviews in the field of energy harvesting describing not only the wide range of possible exploitable materials (namely, lead zirconate titanate (PZT), BaTiO_3_ (BTO), (Na,K,Li)NbO_3_, GaN, zinc oxide (ZnO) nanowires, organic films (poly(vinylidene fluoride); PVDF), cellulose, and a combination of them, all deposited on almost all imaginable substrates) but also the survey of potential devices [11,12,13,14,15,16].

Although inorganic materials have been fairly successfully used in energy harvesting, the high cost, as well as the difficult integrability in a device, hinder their true potential. Further, although inorganic materials possess higher piezoelectric coefficients than polymers, they also exhibit higher elastic moduli (i.e., higher stiffness) [17], which makes them less sensitive to small vibrations and more prone to stress failure [18]. However, polymer-based generators represent a relatively small proportion of the total research field and are almost exclusively based on PVDF and cellulose, which need to be polarized under high electrical fields before use [19,20]. In addition, many of the technologies present in the literature involve long and complex manufacturing processes with high cost/benefit ratios.

Among the inorganic components, ZnO has attracted great attention in recent years, because it possesses several key advantages; among them, there is being a biologically safe piezoelectric semiconductor occurring in a wide range of nanostructured forms. Additionally, it is a potential candidate for commercial purposes, due to its inexpensiveness, relative abundance, and chemical stability in air atmosphere. Further, ZnO nanorods can easily grow in aligned arrays on plastic substrates [21]. Moreover, the use of ZnO dispersed in films has been considered for MEMSs/NEMSs (micro- and nanoelectromechanical systems), sensors, and energy harvesting devices [22].

Some pioneering works have explored the possibility to integrate ZnO spherical nanoparticles (at the maximum filler loading of 20 wt.%) into a UV-curable polymer matrix (namely, SU-8) for producing more flexible devices based on cheap and easy-to-be-scaled technologies [23,24]. All the films showed a good response in terms of root mean square (RMS) voltage measured as a function of the applied waveform, both at low and resonance frequencies, notwithstanding that only spherical nanoparticles were investigated.

It is noteworthy that compared with thermal curing, UV curing is more economical, faster, and environmentally friendly. In fact, it does not involve the use of organic solvents, thus lowering volatile organic compound (VOC) emissions [25,26,27].

Acrylic UV-curable systems undergo radical reactions. Primary radicals are generated through the homolytic scission of the photoinitiator, under exposure to UV radiation; the photoinitiator is added to the reacting system at catalyst concentration (at low weight percentages, usually not exceeding 5 wt.%). These primary radicals propagate the reactions by attacking the acrylic double bonds, thus leading to the formation of propagating macroradicals and finally to the formation of a highly cured 3D network in a very short time (tens of seconds are sufficient).

Based on these considerations, we first prepared UV-cured composite films based on acrylic resin filled with different ZnO morphologies (namely, spherical nanoparticles, bipyramidal structures, flower-like structures, and long needles) [28]. These nanostructures were incorporated at constant loading (4 wt.%) into UV-curable resin. It was found that flower-like morphologies exhibited the best piezoelectric performance both at 150 Hz and at resonance frequency, gaining the maximum normalized root mean square (RMS) voltage of 0.914 mV when 5.79 g acceleration was applied. At variance, ZnO needles showed the lowest RMS voltage values; this finding was interpreted based on the possible direction of the (002) crystallographic planes, which was perpendicular to mechanical solicitation and thus less effective from a piezoelectric point of view.

Supported by these preliminary results, in the present work, we decided to prepare UV-cured composite films made of the same acrylic resin filled with flower-like (ZFL) and needle-like (ZLN) zinc oxide (i.e., corresponding to the highest and lowest piezoelectric performances) in different amounts (i.e., 4, 10, and 20 wt.%). In this way, it was possible to thoroughly study the influence of the filler amount on the piezoelectric response of UV-curable films. In particular, we explored a much lower frequency range (namely, from 1 to 100 Hz), corresponding to the environmental harvesting conditions. This was possible because of the use of a new, reliable piezoelectric apparatus, designed on purpose, which allowed us to work under the aforementioned frequency conditions. Further, instead of RH sputtering, a simpler and, at the same time, effective methodology (i.e., silver painting) was employed for providing the UV-cured composite films with the envisaged electrical conductivity.

The completeness of the UV curing process was assessed by means of FTIR-ATR and DSC analyses. SEM analyses of both film surface and cross-section were performed to evaluate the charge density and homogeneity of distribution of ZnO within the polymer matrix. The piezoelectric behavior of the films was investigated at typical environmental vibration energy for harvesting applications (<100 Hz) and correlated with the mechanical properties of the films measured using dynamic–mechanical analyses.

## 2. Materials and Methods

### 2.1. Materials

Zinc oxide samples were expressly prepared using wet chemistry processes, obtaining powders of two different morphologies: flower-like (ZFL) and needle-like (ZLN) structures [28]. UV-curable resin was commercial bis-phenol A ethoxylate diacrylate (Ebecryl 150; hereinafter coded as EB), kindly supplied by Cytec Industries BV (Vlaardingen, The Netherlands). 2,2-dimethyl-2-hydroxy acetophenone (Irgacure 1173) from BASF (Cesano Maderno-Monza Brianza, Italy) was used as the photoinitiator.

#### 2.1.1. Synthesis of ZnO Morphologies

**Figure 1:**
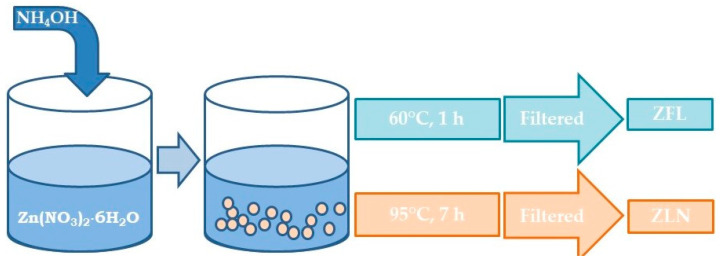
Schematic representation of nanostructured ZnO synthesis. ZFL = ZnO flower-like and ZLN = ZnO needle-like morphologies.

The synthesis of both ZnO forms was performed starting from the same amount of Zn precursor, namely, zinc nitrate hexahydrate (Zn(NO_3_)_2_•6H_2_O; ≥99.0%; purchased from Merck) dissolved in water to reach the solution molarity of 0.05 M. Subsequently, the proper amount of ammonium hydroxide (28%; Carlo Erba Reagents, Italy) was added under continuous stirring for adjusting the solution pH to 10. In the case of flower-like powder, the hydrolysis temperature was 60 °C, and the solution temperature was kept constant for one hour. Needle-like powder was obtained by performing hydrolysis at room temperature and treating the mixture at 95 °C for about 7 h. The ZnO precipitates were filtered, washed several times with deionized water and then with diethyl ether (Merck), and dried in an oven overnight. Figure 1 reports a schematic representation of the synthesis of the two different ZnO morphologies.

#### 2.1.2. Preparation of the Nanocomposite Films

Photoinitiator at 4% wt. (Irgacure 1173) was added to acrylic UV-curable resin; then, ZnO fillers were dispersed in resin and ultrasonicated for 30 min at room temperature. ZnO was dried before being mixed in a vacuum oven for 24 h at 80 °C. The mixing ratios of EB/ZnO (wt/wt) were 100/0, 96/4, 90/10, and 80/20. The dispersions composed of photoinitiator, acrylic resin, and ZnO were coated on glass slides, using a wire wound applicator (nominal thickness: 150 μm) and then exposed to UV radiation provided by a UV oven, namely, an F300 S apparatus (Heraeus Noblelight, USA), working under static conditions. The radiation intensity on the sample surface, measured with a UV meter, was about 800 mW/cm^2^; two not-consecutive exposure periods of 15 s were enough for completing the photopolymerization reaction, also in the presence of the different fillers. The obtained UV-cured films (120 μm thick) were peeled off from the glass substrates and used for the different characterizations and for the fabrication of cantilevers for piezoelectric tests.

### 2.2. Characterization Techniques

#### 2.2.1. Fourier Transform Infrared (FTIR)-Attenuated Total Reflection (ATR) Spectroscopy

The FTIR-ATR spectra of the obtained films were recorded using a Perkin-Elmer FTIR spectrometer (model Paragon 500 equipment) with the aim to assess the completeness of the photopolymerization process. The spectra (32 scans) were recorded in a 4000–400 cm^−1^ wavelength range at 4 cm^−1^ resolution.

#### 2.2.2. Scanning Electron Microscopy (SEM)

The morphology of ZnO powders was investigated using field-emission scanning electron microscopy (FE-SEM), using a Carl Zeiss Sigma microscope equipped with a Schottky field emitter (tip made of <100> tungsten crystal and a ZrO_2_ reservoir). Additionally, a scanning electron microscope (SEM Zeiss Evo 50 XVP with LaB_6_ source) was employed for analyzing the surface and the cross-section of the composite films. SEM analysis was also employed for the observation of the laser cut exploited for preparing cantilevers to be used in the piezoelectric tests. For cross-section observation, the samples were fractured in liquid nitrogen. Backscattered electrons were employed for surface analysis. All the films were gold-metalized using an E5 150 SEM coating unit.

#### 2.2.3. Wide-Angle X-ray Diffraction (WAXD)

Wide-angle X-ray diffraction was carried out using a Philips PW 1830 vertical diffractometer with Bragg–Brentano geometry (Cu Kα radiation, 40 kV, 30 mA). Diffraction patterns of ZnO were collected over the range of 2θ = 10–120°, with steps of 0.02° and 10 s of dwell time and were processed using the Rietveld refinement technique using FullProf software (2011 release) [29]. The average crystallite size was calculated by applying the Scherrer formula [30] on the (101) diffraction peak. Diffraction patterns of the composites were collected under the same conditions as the previous ones, over the range of 2θ = 10–60°.

#### 2.2.4. Differential Scanning Calorimetry

The thermal behavior of the composite films was assessed using a Mettler DSC-822 apparatus. DSC analyses were carried out according to the following cycle: (1) heating from 0 °C to 160 °C at 10 °C/min; (2) cooling down to 0 °C at −10 °C/min; and (3) heating from 0 °C to 160 °C at 10 °C/min. The glass transition temperature (T_g_) was taken at the midpoint of heat capacity changes. Calibration was performed using indium as the standard (T_m_ = 156.4 °C; ΔH_m_ = 28.15 J/g).

#### 2.2.5. Dynamic–Mechanical Analyses

Dynamic–mechanical (DMTA) analyses were performed using DMA Q800 (TA Instruments) in tensile configuration on all the composite films. The following experimental conditions were adopted: temperature range from 25 to 120 °C, heating rate of 3 °C/min, 1 Hz frequency, and 0.05% of oscillation amplitude in strain-controlled mode. Storage modulus (E’) and tanδ curves were recorded. T_g_ values were calculated from the peak value of the tanδ curves. For each formulation, the tests were repeated four times, and the experimental error was calculated as standard deviation for all the measured parameters.

#### 2.2.6. Piezoelectric Measurement Setup

**Figure 2:**
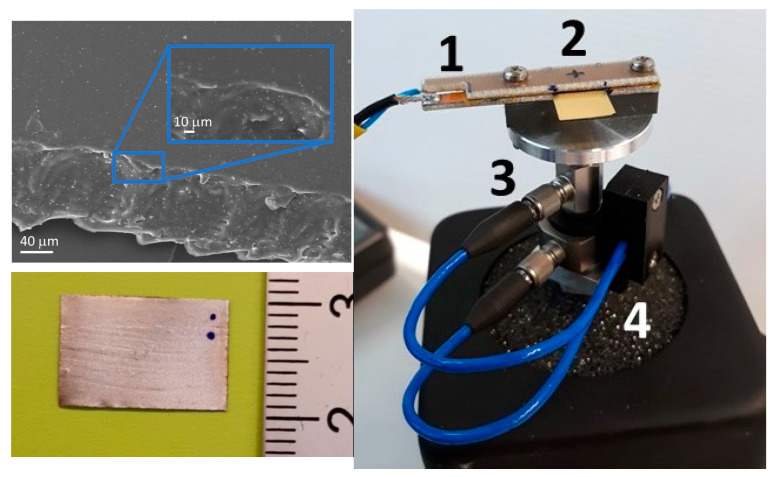
(**Left**, **up**) SEM micrograph of a typical laser-cut EB/ZFL film sample. (**Left**, **down**) Sample of EB/ZFL after the silver painting with a brush. The rectangular part on the left is clamped into the sample holder. (**Right**) View of the system used to clamp the sample under test (1,2) and the two sensors (3,4).

**Figure 3:**
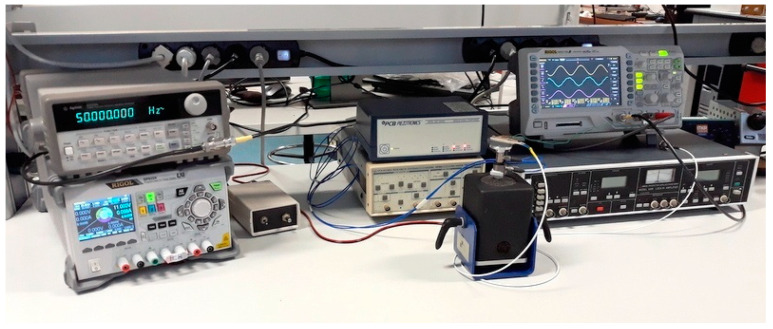
Experimental system for measuring the piezoelectric behavior of EB composite films.

**Figure 4:**
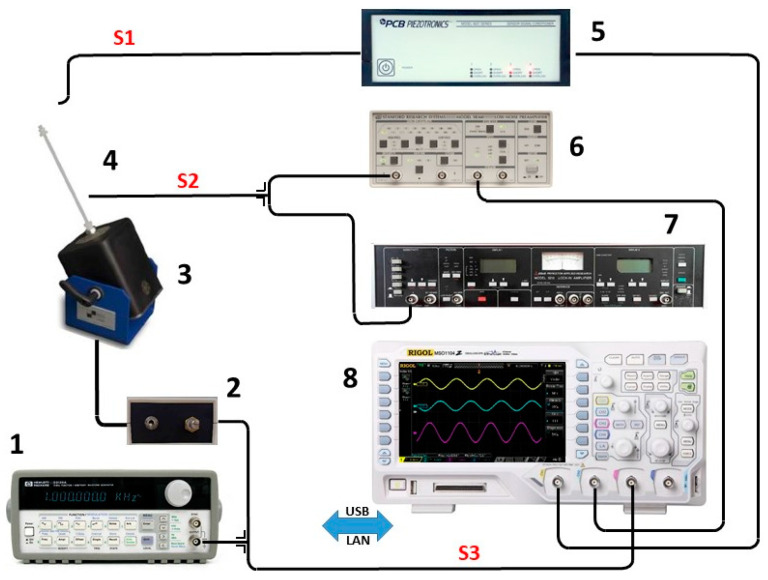
(1) Function generator; (2) audio frequency amplifier; (3) shaker; (4) detector head and sample holder; (5) accelerometer amplifier; (6) low-noise amplifier; (7) lock-in amplifier; (8) oscilloscope. The three signals acquired and processed are: S1—accelerometer signal from the oscillating head; S2—piezoelectric signal generated by the cantilever (DUT); S3—sinusoidal shaker excitation signal with adjustable amplitude and frequency.

Cantilevers (devices under test; DUTs) of about 10 × 15 mm^2^ were fabricated by cutting the UV-cured nanocomposite films using a CO_2_ laser. An SEM image of cut film is reported in Figure 2, evidencing that the laser did not compromise the DUTs. A silver film, working as the electrode to collect the generated charges, was deposited at room temperature on both cantilever sides with a paint brush, using conductive paint. The samples, once positioned in the sample holder, had a free square surface of 1 cm^2^, which was mechanically stressed to obtain piezoelectric signals (Figure 2). All the materials were tested at typical environmental vibration energy of harvesting applications (<100 Hz) using the experimental measurement system shown in Figure 3. The block diagram of the measurement system developed for the electrical measurements is shown in Figure 4. The sinusoidal excitation signal was generated by a programmable function generator (1) at the chosen frequencies and amplitude.

The generator was connected to an audio frequency amplifier (2), which drove the shaker actuator (3) with a power of about 15 W, enough to move the whole test group (4) consisting of the sample itself, the sample holder, and two calibrated sensors, in the full operative frequency range. The original excitation signal was also fed to both the CH 3 input of the oscilloscope (8) for monitoring and synchronization purposes and the lock-in amplifier (7) as a reference signal for demodulation. The CH 1 input of the oscilloscope was connected to the accelerometer amplifier and conditioned the signals from both the accelerometer and the force sensor for the correct measurement. The piezoelectric signal generated by the DUT was connected to a low-noise amplifier (6), equipped with a series of selectable input filters, through a coaxial cable. The amplifier output was connected to the oscilloscope CH 2 input, which clearly showed, at the same frequency, the amplitude and shape of all measured signals. The piezoelectric characterization measurements were performed in a shielded laboratory for electrical measurements at a nominal temperature of 23.0 °C and 50% humidity.

## 3. Results and Discussion

### 3.1. Morphological Analysis of ZnO Powders

**Figure 5:**
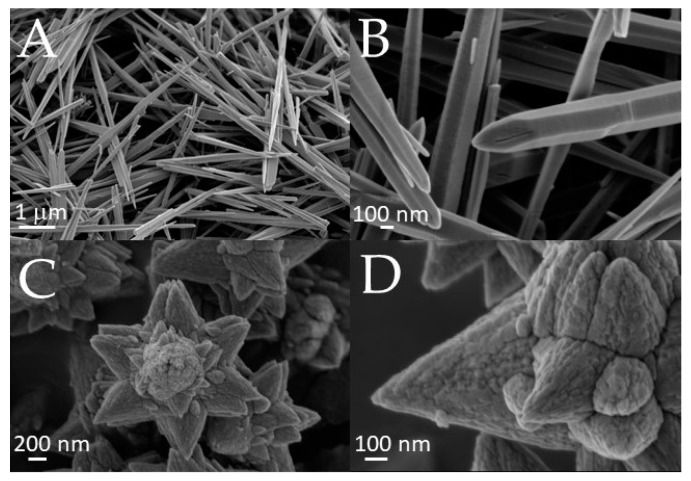
SEM micrographs of ZnO morphologies: (**A**,**B**) long needles (ZLN) and (**C**,**D**) nanoflowers (ZFL).

Typical SEM micrographs of the two different ZnO morphologies considered in this work are shown in Figure 5; different magnifications are reported to highlight the features of each different morphology. The ZLN samples (Figure 5A,B) consisted of bi-dimensional nanocrystals shaped as long needles (about 100 nm wide and 7–8 μm long). ZFL powder was characterized by nanoparticles of about 30 nm (Figure 5D) in size assembled in flowers of 1 μm in size (Figure 5C).

### 3.2. WAXD Analysis of ZnO Nanostructures

**Figure 6:**
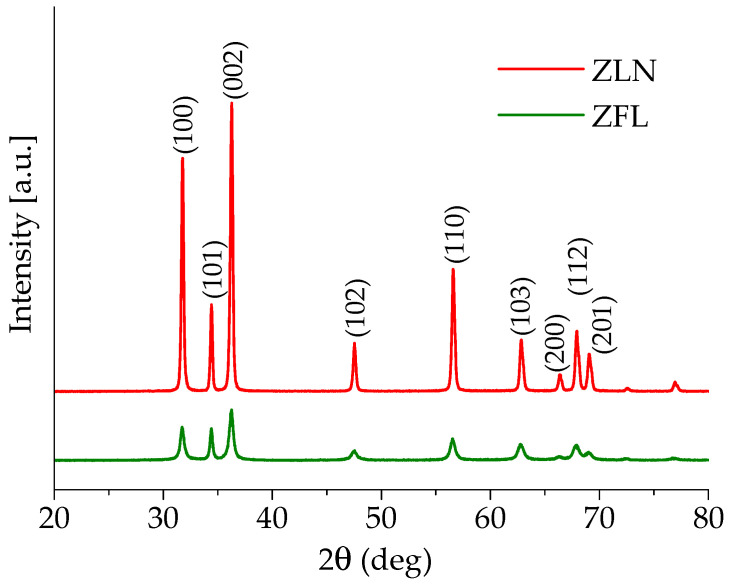
WAXD patterns of needle-like (ZLN) and flower-like nanoparticles (ZFL).

According to the diffraction patterns shown in Figure 6, both synthesized morphologies exhibited a hexagonal wurtzite structure (space group P63mc), irrespective of both the synthesis process and the morphology. The XRD spectra show the typical peaks of zinc oxide, in agreement with those reported in JCPDS card No. 36-1451. The average crystallite sizes, evaluated using the Scherrer formula on the (101) diffraction peaks, resulted to be 30 nm for the ZFL samples and 39 nm for the ZLN samples.

### 3.3. Structural and Morphological Characterization of Composites

#### 3.3.1. Assessment of the Completeness of the UV Curing Process with FTIR-ATR Spectroscopy

**Figure 7:**
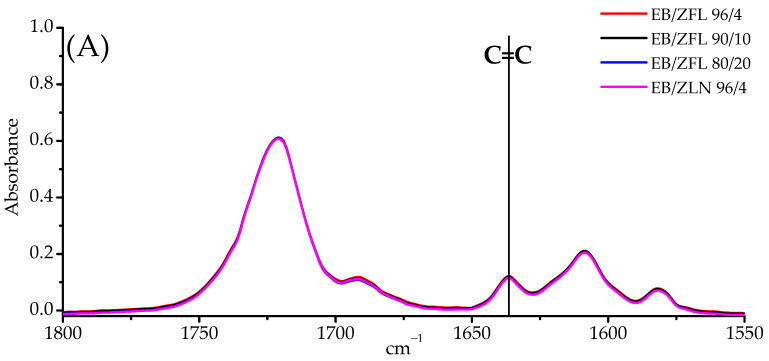

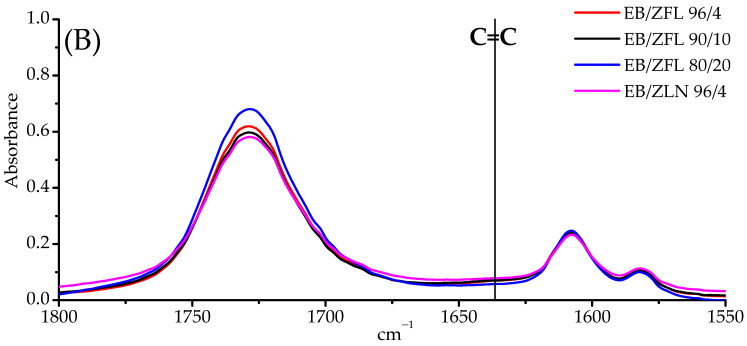
FTIR-ATR spectra of EB-ZnO composites, before (**A**) and after (**B**) exposure to UV radiation. The absence of the band at about 1635 cm^−1^ after exposure to UV radiation is a clear indication of the completeness of the UV curing process.

Ebecryl 150 is a commercially available acrylate with high reactivity, especially in combination with suitable photoinitiators. It is well known that the UV curing of acrylate systems occurs at room temperature, providing the system with the ultimate achievable cross-linking density, i.e., with the maximum conversion of double bonds [31]. However, to assess whether the adopted experimental conditions employed for the photocuring process were suitable for achieving the completeness of the double-bond conversion also at high filler loading, attenuated total reflectance-Fourier transform infrared spectroscopy (ATR- FTIR) was employed. As an example, Figure 7 shows the typical spectra, in the range between 1800 and 1550 cm^−1^, of systems containing ZLN and ZFL morphologies (ZFL in different amounts), before (Figure 7A) and after (Figure 7B) exposure to UV radiation.

In all cases, the complete disappearance of the band at 1635 cm^−1^ is evident, indicating that all the acrylic double bonds [32] participated in the curing reaction. This finding, as reported in the next paragraph, is further supported by the absence of exothermal peaks in the first-heating-stage DSC thermogram traces.

#### 3.3.2. DSC Characterization of Composite Films

**Figure 8:**
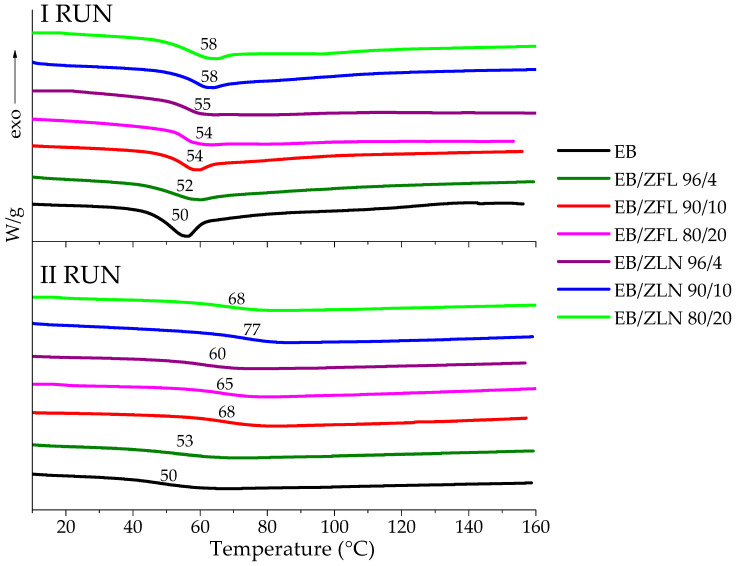
DSC thermograms (1st and 2nd runs) of ZnO composite films (heating rate: 10 °C/min). The arrow indicates the direction of the exothermic flux.

DSC measurements were performed to both confirm the completeness of the curing process and to investigate the influence of fillers on the glass transition temperature of the EB matrix. In Figure 8, the first and second heating DSC runs for the ZFL and ZLN composite films are presented. For all the samples, in the first run, the enthalpy relaxation superimposed on the specific heat changes associated with the glass transition of the polymer was ascribed to the fast UV curing process used for preparing the composite films; the process freezes the macromolecules in a non-equilibrium thermodynamic state. Furthermore, in the first run, no exothermic phenomena were observed aside from enthalpy relaxation, thus indicating that the completeness of the curing process was achieved under the adopted experimental conditions, even in the presence of different fillers and different filler loading levels. The different amounts of filler did not significantly modify the T_g_ (I RUN) of resin (i.e., T_g_ = 52 °C, 54, and 54 °C for EB/ZFL 96/4, EB/ZFL 90/10, and EB/ZFL 80/20; T_g_ = 55 °C, 58, and 58 °C for EB/ZLN 96/4, EB/ZLN 10, and EB/ZLN 20, respectively).

In the second run, enthalpy relaxation disappeared, and only glass transition was detectable, which was higher than that in the first run. All the composites had significantly higher T_g_ than unfilled resin, indicating that the fillers limited the mobility of the EB macromolecules, which re-arranged in a more stable conformational structure after the first heating-and-cooling cycle [33].

#### 3.3.3. Morphological Analysis of EB-ZnO Surfaces

**Figure 9:**
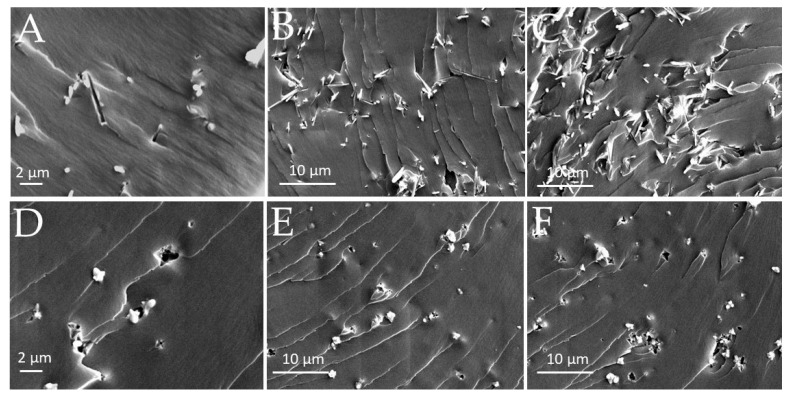
Typical SEM micrographs of fracture surfaces of EB/ZFL 96/4 (**A**), EB/ZFL 90/10 (**B**), EB/ZFL 80/20 (**C**), EB/ZLN 96/4 (**D**), EB/ZLN 90/10 (**E**), and EB/ZLN 80/20 (**F**).

The SEM micrographs of cryogenically fractured surfaces of ZnO composite films are shown in Figure 9. Different magnifications are reported to evidence the morphological features of each composite. In both series of ZnO composites, ZLN and ZFL particles maintained their morphology and showed an almost uniform distribution within the polymer matrix. By increasing the filler amount, the number of aggregates increased. In some points, both ZLN and ZFL fillers appeared not to be perfectly embedded in the polymer matrix (evidencing the presence of voids) and/or to have been pulled out during fracturing, indicating poor interaction with acrylic resin.

**Figure 10:**
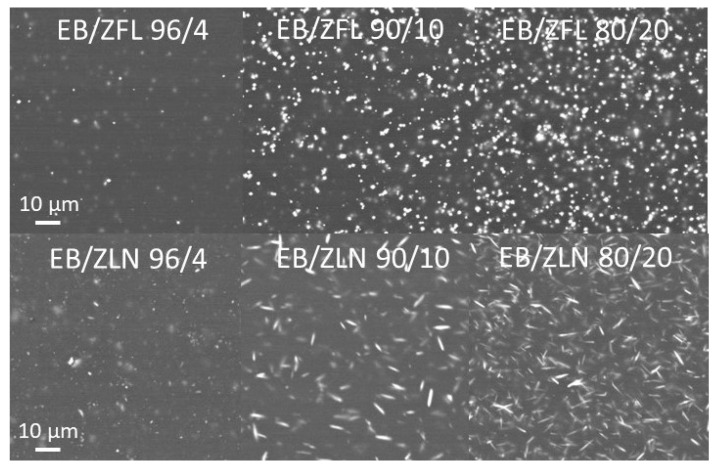
SEM micrographs obtained using backscattered electrons of EB/ZFL and EB/ZLN 96/4, 90/10, and 80/20 surfaces.

The piezoelectric response of composites, which will be discussed in the next section, could be interpreted in terms of the piezoelectric element density present on the surface of the films. For this reason, the SEM micrographs of ZFL and ZLN composite surfaces containing 4, 10, and 20 wt.% of filler are compared in Figure 10. It is clear that, independently from the analyzed morphology, all the composite surfaces showed a good distribution of piezoelectric filler, and that the element density increased with the increase in filler loading.

#### 3.3.4. Structural Analysis of ZnO Composites

**Figure 11:**
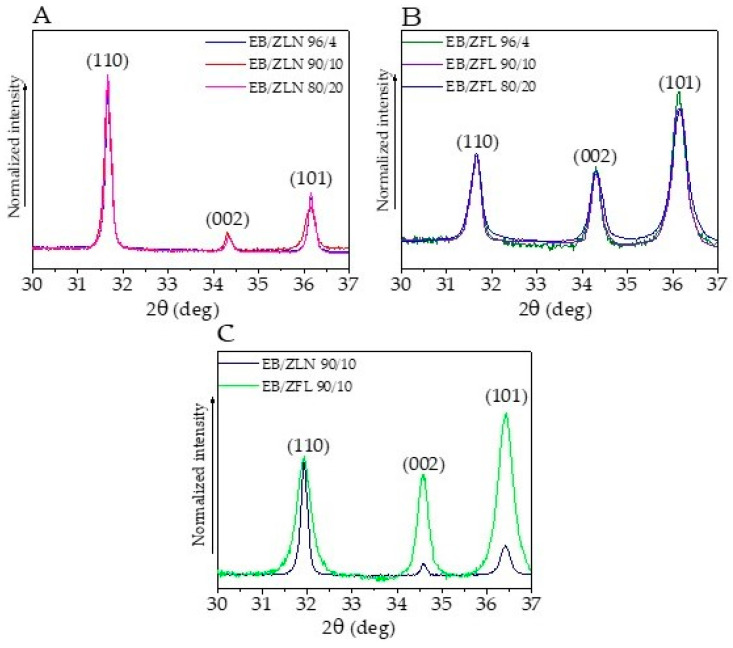
The typical WAXD patterns of ZnO composites in the range between 30 and 37° highlight the preferential orientation of crystallographic planes of ZFL and ZLN particles in the composite films. Spectra were normalized with respect to the (110) crystallographic peak. Spectra of EB/ZLN (**A**) and EB/ZFL (**B**) composites at different filler loadings. Comparison of EB/ZLN and EB/ZFL 90/10 spectra (**C**).

The X-ray diffraction patterns of ZnO composites were characterized by the presence of the broad amorphous halo centered at about 2θ = 19° due to the acrylic polymer matrix. The most interesting result provided by these measurements is the different orientation of the characteristic crystallographic plane of ZFL and ZLN in the composite films. In Figure 11A,B, the diffraction patterns of ZLN and ZFL composites, respectively, at different filler loading levels, are reported in the range between 30 and 37°. The curves were normalized with respect to the (110) crystallographic peak. As expected, no variations in the relative intensity of the peaks occurred by changing the ZnO loading. Furthermore, by comparing ZFL and ZLN composites (Figure 11C), it is noteworthy that the composite containing ZLN had a low amount of (002) planes oriented according to the plane of the film, whereas these planes were more abundant in the films incorporating ZFL. This result is important because it could help to understand the different behavior in terms of piezoelectric response.

#### 3.3.5. Dynamical–Mechanical Thermal Analyses of EB Composite Films

**Figure 12:**
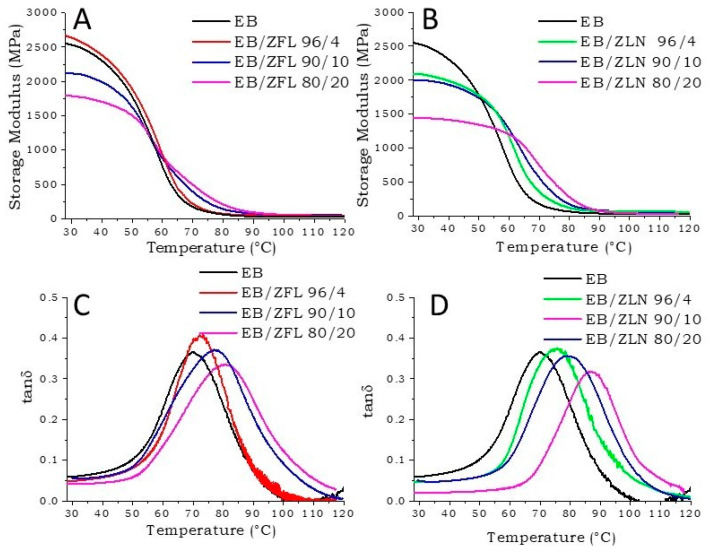
Storage modulus of EB/ZFL (**A**) and EB/ZLN (**B**) composite films containing different amounts of ZnO. Tanδ of EB/ZFL (**C**) and EB/ZLN (**D**) composite films containing different amounts of ZnO.

Figure 12A,C show the storage modulus and tanδ of EB composites with different amounts of ZFL, respectively. From a general point of view, it is possible to observe that by increasing the ZFL loading, (i) the storage modulus (Figure 12A) in the glassy state decreased, probably due to the presence of poor interaction and/or voids (also evidenced by SEM analyses) between polymer chains and fillers, and (ii) T_g_ increased (peak of tanδ in Figure 12C), because as already observed using DSC, ZnO acted as a reinforcement of the polymer network [34,35]. Further, the tanδ curves broadened due to restriction effects, suggesting a slight suppression of the glass transition by the presence of ZnO particles [36].

The same behavior was shown by EB/ZLN composites, as shown in Figure 12B,D. However, the composites containing ZLN filler had higher T_g_ and lower storage modulus in the glassy state than those containing ZFL at equal filler loading (Table 1). The most remarkable effect of ZLN with respect to ZFL particles can be probably ascribed to the different sizes of the two fillers; in fact, ZLN nanoparticles were about 100 nm wide and 7–8 μm long, while ZFL particles were 1 µm large.

#### 3.3.6. Piezoelectric Behavior at Low Frequencies (≤100 Hz)

**Figure 13:**
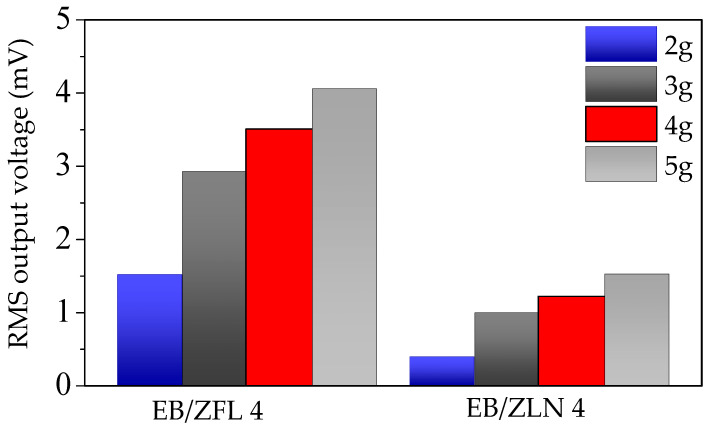
RMS output voltage values produced by composites containing 4 wt.% of ZnO (flower-like (ZFL) and needle (ZLN) structures) at the frequency of 19 Hz and different acceleration values (namely, 2, 3, 4, and 5 g).

The effective piezoelectric properties of composite films were examined with a specific investigation on similar samples made of unfilled UV-cured resin. The generated voltages vs. excitation frequency were normalized to maximum acceleration, and the obtained values were elaborated as root mean square (RMS) to compare the results with the filled systems. The normalized generated voltages of EB film at low frequencies (below 100 Hz) were close to the noise limit, without any correlation with the excitation frequency. This result clearly indicates that bare resin is not able to generate any electrical signal when solicited at frequencies below 100 Hz, as already found at higher frequencies and acceleration values [28]. Conversely, EB/ZnO composite films displayed enhanced generation properties. Figure 13 shows the normalized voltage RMS values (of samples containing 4 wt.% of ZnO) at 19 Hz and different accelerations (2, 3, 4, and 5 g) as an example.

**Figure 14:**
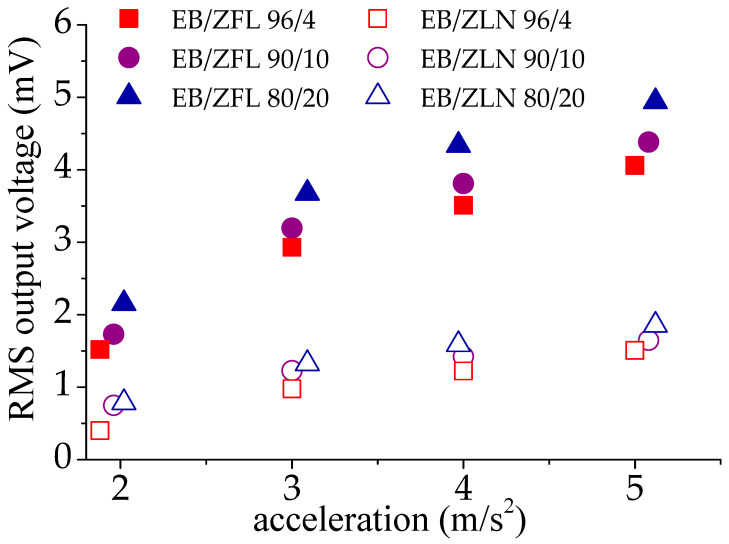
RMS output voltage of EB-ZFL samples with different ZFL amounts at the frequency of 19 Hz vs. the acceleration value.

The highest RMS voltage was registered in EB/ZFL across all the range of analyzed frequencies and irrespective of the loading. To explain the different particle behavior, it is important to consider that the piezoelectric effect of ZnO is linked to the orientation of the (002) crystallographic planes; if these planes are oriented perpendicularly to the applied solicitation, piezoelectricity can be detected [37]. In EB/ZFL films, even if ZFL particles have random orientations, their peculiar geometry justifies the possibility that a portion of (002) planes are oriented in the desired direction, thus giving rise to voltage generation upon solicitation. Conversely, in EB/ZLN films, the z-direction of needles mainly lies on the plane of the film (as also evidenced by the SEM images shown in Figure 9 and Figure 10). This means that (002) planes, being perpendicular to their z-direction [38,39], are mainly parallel to mechanical solicitation, thus determining lower RMS voltages. The increase in ZnO positively affects the piezoelectric response of the composites; as shown in Figure 14, the RMS voltage generated at 19 Hz as a function of the acceleration increased in the presence of the highest amount of ZFL and ZLN used (i.e., 20 wt.%). However, the increase was not proportional to the amount of filler and was even worse for ZLN composites.

These results do not depend on the dispersion of filler or on the number of particles, which increased with the increase in filler loading, as observed using SEM (Figure 10). The piezoelectric response as a function of the filler amount seems more linked to the decrease in storage modulus of the composites at higher ZnO concentrations. The presence of voids and poor interaction between filler and matrix was probably responsible for the reduction in the crystallographic distortion induced in ZnO particles by mechanical solicitation during the tests.

Finally, it is worthy to underline that despite the large gap between the measured voltage values in this work and those related to fully inorganic piezoelectric systems (which are two–three orders of magnitude higher) [38,39], the proposed piezoelectric films show two main advantages, i.e., flexibility and cost-effective scalability, which represent key elements in the design and development of innovative devices in the field of green technologies [40,41,42].

## 4. Conclusions

ZnO in the shape of flowers (ZFL) and needles (ZLN) was synthesized and embedded into UV-curable acrylic resin (EB), with the aim to study the effect of filler loading on the piezoelectric properties of the resulting composite films. As a result, 120 μm thick films were obtained with the highest achievable cross-linking density.

The composites showed uniform dispersion of fillers within the polymer matrix. However, by increasing the filler amount, the number of aggregates increased. In some points, both ZLN and ZFL fillers appeared not to be perfectly embedded in the polymer matrix (presence of voids) and/or to have been pulled out during fracturing, indicating poor interaction with acrylic resin.

By increasing the filler content, the glass transition temperature (T_g_) increased, and the storage modulus in the glassy state decreased. The piezoelectric response generated by the polymer composite films was good in terms of RMS (root mean squared) voltage measured at 19 Hz as a function of the acceleration; it was higher when the highest amount of ZFL was used (i.e., 20 wt.%), though the increase was not proportional to the filler loading. This did not depend on the dispersion of filler or on the number of particles on the surface. The piezo-behavior seemed more related to the decrease in storage modulus of the composites at high ZnO loading.

## Figures and Tables

**Table 1:** T_g_ and storage modulus at 30 °C of EB and its composites.

	T_g_	E’ @ 30 °C
EB	70 ± 2	2537 ± 52
EB/ZFL 96/4	73 ± 1	2637 ± 62
EB/ZFL 90/10	78 ± 3	2111 ± 87
EB/ZFL 80/20	81 ± 3	1791 ± 110
EB/ZLN 96/4	75 ± 2	2089 ± 49
EB/ZLN 90/10	80 ± 2	1984 ± 64
EB/ZLN 80/20	86 ± 1	1439 ± 74

## Data Availability

Not applicable.
